# Religious pilgrimage and COVID-19. An observational report in Italy from contact tracing activities: Emanuela Maria Frisicale

**DOI:** 10.1093/eurpub/ckac131.084

**Published:** 2022-10-25

**Authors:** F Ferraro, EM Frisicale, A Rapiti, C Marotta, S Bonfigli, U Angeloni, F Maraglino, G Rezza

**Affiliations:** DG Health Prevention, Ministry of Health, Rome, Italy

## Abstract

**Issue/Problem:**

Religious Mass Gathering (MG) represent one of public health challenges for Health Authorities due to potential spread of communicable diseases. This is much more true during a pandemic as COVID-19. Surveillance is crucial to prevent further spreading of infectious disease related to a religious MG.

**Description of the problem:**

During international contact tracing activities an increase of reporting of COVID-cases with a travel history to a Catholic shrine in Europe was observed, despite travel restrictions put in place. In order to promote public health actions as risk communication, a risk evaluation was conducted. A descriptive analysis was carried out: personal and vaccination data were collected; for cases, date and type of positive tests, date of symptoms’ onset were collected; for high-risk contacts, date and type of negative tests at the end of follow-up were collected. Frequencies were calculated.

**Results:**

Six journeys back from Medjugorje were identified, with at least one positive case. All trips took place between 18/09/2021 and 29/10/2021. 31 positive cases out of 160 travellers were identified, with number of cases per travel ranging from 1 to 11.

**Lessons:**

Religious MG represent an important global health issue. Even though a specific surveillance was not activated, international contact tracing activities turned out a great source of epidemic intelligence and consequent surveillance and control activities led to risk assessment and communication actions.

**Key messages:**

• In the pandemic context, where travel restrictions were put in place, surveillance for Religious MG should be always implemented.

• Cooperation among all the stakeholders involved as Church, travel agency, Regional Health Systems and Government Bodies has to be promoted for specific surveillance in religious MG events.

